# High-Sucrose Diet Exposure on Larvae Contributes to Adult Fecundity and Insecticide Tolerance in the Oriental Fruit Fly, *Bactrocera dorsalis* (Hendel)

**DOI:** 10.3390/insects14050407

**Published:** 2023-04-24

**Authors:** Lei Wang, Dan-Dan Wei, Gui-Qiang Wang, Han-Qin Huang, Jin-Jun Wang

**Affiliations:** 1 Key Laboratory of Entomology and Pest Control Engineering, College of Plant Protection, Southwest University, Chongqing 400715, China; 2 Key Laboratory of Agricultural Biosafety and Green Production of Upper Yangtze River (Ministry of Education), Academy of Agricultural Sciences, Southwest University, Chongqing 400716, China

**Keywords:** Tephritidae, life history traits, transcriptome, gene expression, plasticity

## Abstract

**Simple Summary:**

The oriental fruit fly, *Bactrocera dorsalis*, is a highly significant invasive pest in agriculture, causing damage to over 600 species of fruits and vegetables. To gain a better understanding of its host adaptation and rapid spread, it is crucial to investigate the effects of changes in nutrient content on the phenotype and gene expression of *B. dorsalis*. In this study, we examined the developmental duration, adult fecundity, insecticide susceptibility, and gene expression patterns of *B. dorsalis* by varying the concentration of sucrose in its larval diet. Our findings indicate that a high-sucrose diet during larval stages resulted in a longer developmental period, higher adult fecundity, and greater tolerance to malathion. In contrast, a low-sucrose diet led to smaller body size, shorter developmental duration, and higher sensitivity to beta-cypermethrin. Additionally, we identified differentially expressed genes associated with various metabolisms, hormone synthesis and signaling, and immune-related pathways under different sucrose concentrations in the larval diet. These results suggest that dietary sucrose plays a significant role in phenotypic adjustments and gene expression patterns in *B. dorsalis*.

**Abstract:**

*Bactrocera dorsalis* (Hendel) (Diptera: Tephritidae) is one of the broad host ranges and economically-important insect pests in tropical and subtropical areas. A wide range of hosts means they have strong adaptation ability to changes in dietary macronutrients (e.g., sucrose and protein). However, the effects of dietary conditions on the phenotypes and genotypes of *B. dorsalis* are still unclear. In this study, we aimed to investigate the effects of larval dietary sucrose on the life history traits and stress tolerance of *B. dorsalis*, and its defense response at the molecular level. The results showed that low-sucrose (LS) induced decreased body size, shortened developmental duration, and enhanced sensitivity to beta-cypermethrin. Otherwise, high-sucrose (HS) diet increased developmental duration, adult fecundity, and tolerance to malathion. Based on transcriptome data, 258 and 904 differentially expressed genes (DEGs) were identified in the NS (control) versus LS groups, and NS versus HS groups, respectively. These yielded DEGs were relevant to multiple specific metabolisms, hormone synthesis and signaling, and immune-related pathways. Our study will provide biological and molecular perspective to understand phenotypic adjustments to diets and the strong host adaptability in oriental fruit flies.

## 1. Introduction

Sugars are the major energy source for insects to maintain the basic activities required for life. In many Dipteran flies, diets containing sucrose and yeast hydrolysates (the main source of proteins) have been proven to be more conducive to reproductive development and fertility enhancement than sucrose-only diets [1,2,3,4]. Dietary restriction (changes in the ratio of yeast and sucrose) has also been confirmed in the oriental fruit fly, *Bactrocera dorsalis* (Hendel), and protein and sucrose are two important nutritional sources in the growth and development of this fly [5]. A previous study has shown that different adult diets have great effects on the fecundity of female adults and the hatching rate of eggs, especially the changes in protein concentration in adult diets [6,7].

The larval stage is a critical period affecting the growth of insects, which is directly related to the later development of individuals, and determines the individual adult body size. The larval stage is also the most important feeding stage of holometabolous insects, which provides nutrients and energy for their survival, growth, and metamorphosis [8,9]. The increase in food consumption at the late-instar larval stage can help them get enough nutrients for successful pupation and adult emergence. During the growth and development of insects, the lack of food and nutrition directly affects their growth, metamorphosis, and lifespan [9,10,11]. Under sufficient food conditions, insects store excess energy in fat [12,13]. Once food shortages occur, stored lipids can be quickly metabolized and converted into energy substances under the regulation of multiple hormones [14]. However, the molecular mechanisms underlying the high- and low-sucrose concentration diets imposed on the oriental fruit fly larvae remain unknown. Furthermore, whether the sucrose stress on the larvae will affect the fitness of the subsequent adults remains unclear.

The oriental fruit fly is one of the most important invasive species in China that attacks a diversity of fruits and vegetables. As a holometabolous insect, the damage caused by *B. dorsalis* occurs through oviposition punctures and subsequent larval feeding in fruits. Therefore, the nutritional conditions at the larval stage are critical for the development and reproduction of these flies. Indeed, adults tend to be more resistant to food-limited stress due to their strong dispersal ability, and whether the larval food stress at the larval stage affects the ecological fitness of adults has not been well studied.

By focusing on the strong adaptability of *B. dorsalis* to different host plants that may contain varied levels of sucrose, we hypothesized that the changes in sucrose concentrations in food could affect the life history traits and adaptive capacity of the oriental fruit fly, *B. dorsalis*. Thus, we examined the effects of different dietary sucrose concentrations on the growth and development of *B. dorsalis* larvae, as well as female fertility and insecticide susceptibility. Moreover, we also investigated the influence of sucrose on *B. dorsalis* in gene expression using the RNA-seq technique. Our study will provide reference for a better understanding of the effect of sucrose on phenotypic adjustments in *B. dorsalis* at both organismal and molecular levels.

## 2. Materials and Methods

### 2.1. Insects

The test insects were originally collected from infested fruits in citrus orchards in Hainan Province, China, in 2008. The insects were maintained in the laboratory for more than 100 generations. The larvae were fed on an artificial diet consisting of yeast powder, sucrose, corn and wheat flour, agar, sorbic acid, vitamin C, and linoleic acid, and the adult flies were fed on an artificial diet consisting of sucrose, yeast hydrolysate, vitamin C, and water [15]. Different developmental stages of *B. dorsalis* were kept in a temperature-controlled insectary at 27 °C and 70% relative humidity, with a 14 h:10 h (L:D) photoperiod.

### 2.2. Experimental Diets

The experimental diets of larvae used in this study were slightly modified from the previous study [7], and we varied the amount of sucrose extract in the larvae diet into three groups. Based on the normal culture conditions in the laboratory, 12.0% sucrose (NS) was used as the control [15], and two sucrose concentrations, i.e., 8.0% sucrose and 16.0% sucrose, were set as the low-sucrose (LS) and high-sucrose (HS) conditions, respectively. The sucrose content configuration is shown in Table S1, and the percentage of sucrose was measured by an LB-32T refractometer (Su Wei, Guangdong, China). The adults were fed a normal diet, as previously mentioned.

### 2.3. Effect of Diets on Development

Freshly-laid eggs were collected in 1.5 mL centrifugal tubes filled with orange juice, and incubated in a temperature-controlled insectary as mentioned before. Newly emerged larvae were transferred into 100 mm petri dishes (40 flies per dish, 3 dishes per treatment) filled with the experimental diets. When the late-third instar larvae jumped into the sand basin, the pupation was regarded as the end of the larval stage. The time from egg hatching to pupation (120 flies per treatment) was recorded as the developmental period of the larvae of *B. dorsalis*. The pre-pupa on the day of pupation was screened out with a sieve pot daily, and placed in a special plastic cage. The bottom of the plastic cup was covered with a layer of wet fine sand about 3 cm thick, and the top of the plastic cup was sealed with gauze until adult emergence. The time from pupation to eclosion (n_LS_ = 76, n_NS_ = 99, and n_HS_ = 97; n is the number of flies) was recorded as the developmental period of pupae. The pupation rate was calculated by comparing the total number of pupae to the total number of larvae in the vial after all insects had pupated. Additionally, the pupae were individually weighed to calculate the average pupal weight under different sucrose concentrations (n_LS_ = 76, n_NS_ = 99, and n_HS_ = 97).

For the eclosion, pupae were placed into plastic cages at around 12 h post-pupation. The emergence rate was calculated by comparing the total number of adults to the total number of pupae in the vial after all insect emergences. Adults that eclosed on the same day were placed in a common cage and fed on an artificial adult diet as previously stated. Adult sexual maturity was considered when eggs laid by females appeared on the wall of the cage. The time from emergence to adult oviposition was recorded as the developmental period of pre-oviposition. In all treatments, three replications were conducted, with 40 insects in each replicate.

### 2.4. Effect of Diets on Ovary Development and Fecundity

Adults were placed into jars (100 mm high × 70 mm diameter), and 5 jars were set up for each of the 3 diets (a total of 15 jars). Ten females (*n* = 50 females per diet) and ten males were placed in each jar. Food was placed in a small container and replaced daily. To assess ovary development, 9-day-old virgin female flies (*n* = 30 females per diet) were randomly removed for dissection, and the surface area of individual ovaries was determined. A Leica M205A stereomicroscope (Leica Microsystems, Wetzlar, Germany) was used to capture the images.

Then, 9-day-old female adults were mated with males (fed on normal larval food) in the jar. To investigate the fecundity of female flies, 1.5 mL centrifugal tubes filled with orange juice were used to induce females to lay eggs, and the tubes were replaced daily. The number of eggs was counted from 4 pm to 5 pm every day from 11 to 17 days. Tests on ten pairs of male and female insects per treatment per day were conducted.

### 2.5. Bioassay of Malathion and Beta Cypermethrin

Two insecticides, i.e., malathion (organophosphorus) and beta deltamethrin (pyrethroid), are commonly used for control of *B. dorsalis* in the field. Therefore, we assessed changes in susceptibility to malathion (98.5% purity) and beta cypermethrin (95.0% purity, Chem Service, West Chester, PA, USA) in adult flies following treatment with different concentrations of sucrose on their corresponding larvae. LD_25_ (malathion 150 mg/L; beta cypermethrin 110 mg/L), LD_50_ (malathion 200 mg/L; beta cypermethrin 150 mg/L), and LD_75_ (malathion 250 mg/L; beta cypermethrin 190 mg/L) of these insecticides were applied to the pronotum of 5-day old adults by a PB600-1 Repeating Dispenser (Hamilton Company, Reno, NV, USA) [15]. The mortality of *B. dorsalis* was then calculated at 24 h post-treatment. Control flies were treated with the same amount of acetone only. Three replications were conducted for all the treatments, and 30 insects were used in each replicate.

### 2.6. RNA Sequencing

The total RNA was isolated from 5-day-old female adults by using the TRIzol reagent (Life Technologies, Carlsbad, CA, USA). RNA purity and concentration were detected by a NanoVue UV-Vis spectrophotometer (GE Healthcare Biosciences, Uppsala, Sweden). About 6 flies from each sample of LS, NS, and HS groups were collected with three biological replicates. To build sequencing libraries, the Illumina TruSeqTM RNA sample prep kit (Illumina Inc, San Diego, CA, USA) was used, and the libraries were sequenced with paired-end technology using the Novaseq 6000 platform by BioMark Company (Wuhan, China). The original raw reads in fastq format were filtered to remove contaminating adapter fragments, reads containing more than 5% ‘N’, and low-quality reads. Simultaneously, Q20 (the proportion of reads with quality value ≥ 20) and Q30 (the proportion of reads with quality value ≥ 30) of the clean reads were calculated. The clean reads were then mapped to the reference genome sequence of *B. dorsalis* (ASM78921v2, GenBank assembly accession: GCA_000789215.2) using Hisat2 (http://ccb.jhu.edu/software/hisat2/index.shtml, accessed on 3 November 2020). Novel transcripts and genes were achieved by StringTie 2.1.2 (https://ccb.jhu.edu/software/stringtie/index.shtml, accessed on 3 November 2020) on the base of the reference genome.

### 2.7. Differential Gene Expression and Functional Annotation Analyses

In this study, the TPM (Transcripts Per Kilobase of exon model per million mapped reads) approach was used to investigate the expression levels of genes [16]. Differential expression genes between the LS vs. NS and the HS vs. NS were analyzed using DESeq2 software [17]. The BH (Benjamini and Hochberg) approaches were performed to obtain the adjusted *p* values with the controlling of the false discovery rate (FDR). The absolute value of log_2_ (fold change) ≥ 1 and adjusted *p*-value < 0.05 were used as the threshold to judge significant differences in gene expression. Furthermore, the KOBAS and Goatools software programs were used for the Kyoto Encyclopedia of Genes and Genomes (KEGG) and Gene Ontology (GO) enrichment analyses, respectively [18,19].

### 2.8. qPCR Detection

Based on DEGs, 7 genes in the insulin signal pathway were selected for validation by qPCR. The qPCR primer is described in Table S2. As previously mentioned, total RNA was isolated from 5-day-old adults. After RNA extraction, genomic DNA was removed using DNase I (Promega, Madison, WI, USA). The cDNA was synthesized with PrimeScript™ 1st Strand cDNA Synthesis Kit (TaKaRa, Dalian, China). Then, qPCR was conducted using NovoStart^®^ SYBR qPCR SuperMix (Novoprotein, Shanghai, China) by a CFX96 instrument (Bio-Rad, Singapore), as described previously [20]. The α-tubulin gene (GU269902) was selected as an internal reference gene [21]. The relative expression levels were calculated using qBase [22]. Each qPCR consisted of three biological replicates.

### 2.9. Statistical Analysis

The data were analyzed using SPSS Statistics version 20 software (SPSS Inc., Chicago, IL, USA). The data are presented as mean ± standard error (SE). Student’s *t*-test (*p* < 0.05) was used to determine differences between the means of ovarian surface area and fecundity. Periods, pupal weight, pupation, and eclosion rates were statistically analyzed using one-way ANOVA followed by Tukey’s Honestly Significant Difference tests (significance level: *p* < 0.05). All figures were produced in GraphPad Prism 8.0 software (GraphPad Software Inc., San Diego, CA, USA).

## 3. Results

### 3.1. Biological Parameters of B. dorsalis under Different Sucrose Concentration Diets

Feeding larvae with different concentrations of sucrose diets affects the developmental duration of *B. dorsalis* (Table 1). Because the flies in the control group were a domesticated population that had adapted to artificial diets, we tended to focus on the effects of the changed sucrose contents in the larval diets imposed on the biological parameters when compared with the standard sucrose contents (control group). The developmental duration of larvae was prolonged with the increase in sucrose concentration in diets. There was a significant difference between the LS and NS groups (*p* < 0.05). The developmental duration of *B. dorsalis* larvae fed with a low-sucrose concentration diet was the shortest (10.62 ± 1.306 days), and the HS group was the longest (12.24 ± 1.337 days). In terms of the developmental duration of pupae, it was also slightly prolonged with the increase in sucrose concentration, but the differences were not significantly different. The pre-oviposition period also showed an extension trend as sucrose concentration increased. The pre-oviposition period of the HS group was significantly longer than that of the NS group (*p* < 0.05), while there was no significant difference between the LS group and the NS group.

The pupation rate, pupal weight, and other biological parameters of *B. dorsalis* were also affected by sucrose in the diets of larvae. Compared with the NS group, both the HS group and LS group showed a decreasing trend in the pupation rate, pupal weight, and eclosion rate. In terms of pupation rate, a significant difference was detected between the LS group and the NS group (*p* < 0.05), but there was no significant difference between the HS group and the NS group. Among them, the pupation rate of the larvae in the LS group was the lowest (63.33%), followed by the HS group (80.83%), and the NS group was the highest (82.50%). For pupal weight, there were significant differences between the NS group and the HS group (*p* < 0.05), and between the NS group and LS group (*p* < 0.05). However, there was no significant difference in eclosion rate among the LS, NS, and HS groups.

### 3.2. Fecundity and Development of the Ovary under Different Concentrations of Sucrose Diets

**Figure 1:**
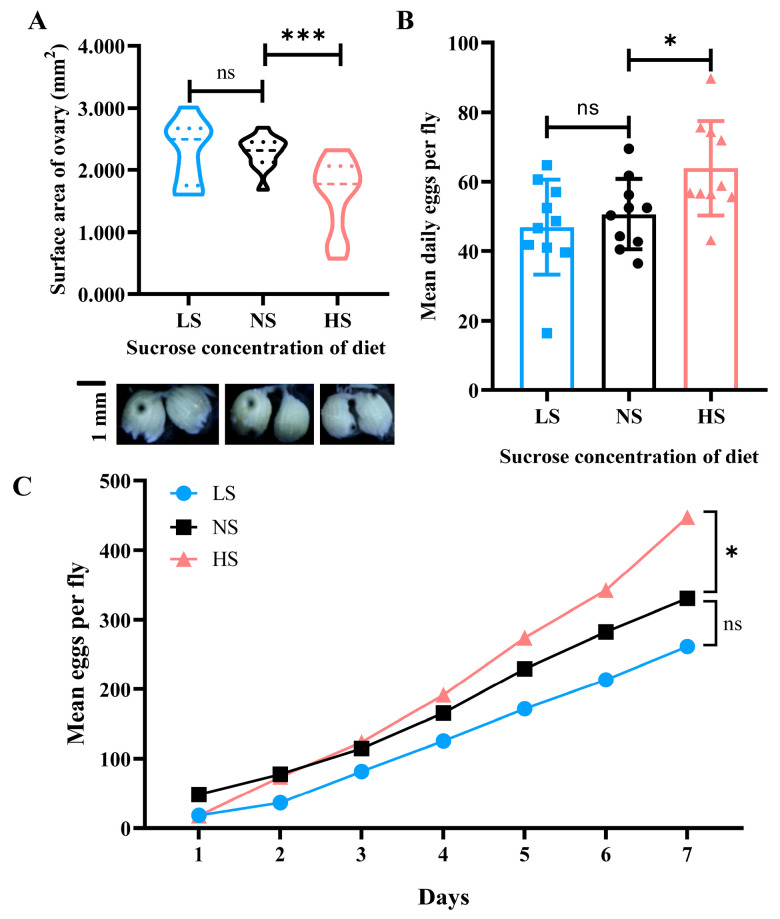
The surface area of the adult ovary on day 9 and the egg-laying amount for 7 consecutive days under different sucrose concentrations mediated by larval diets. (**A**) Adult ovary surface area distribution on day 9. Pictures of the ovary were captured on day 9; (**B**) The average daily egg production of each female adult at the age of 11–17 days; (**C**) The cumulative egg production of each female adult at the age of 11–17 days. NS, normal sucrose; LS, low-sucrose; HS, high-sucrose. *** denotes significance at *p* < 0.001, * denotes significance at *p* < 0.05, and ns denotes no significance.

The ovary size of *B. dorsalis* in the HS group (*t* = 7.627; df = 82; *p* = 0.0006) was significantly smaller than that in the NS group (Figure 1A). However, there was no obvious change in the ovary size in *B. dorsalis* between the LS and NS groups. In the HS group, the cumulative (*t* = 2.472; df = 18; *p* = 0.0237) and average daily (*t* = 2.198; df = 18; *p* = 0.0430) egg production per adult of *B. dorsalis* was significantly increased when compared to the NS group (Figure 1B,C), while there was no significant change in egg laying amount between the LS and NS groups.

### 3.3. The Susceptibility of B. dorsalis to Insecticides in Different Sucrose Diet Concentrations

**Figure 2:**
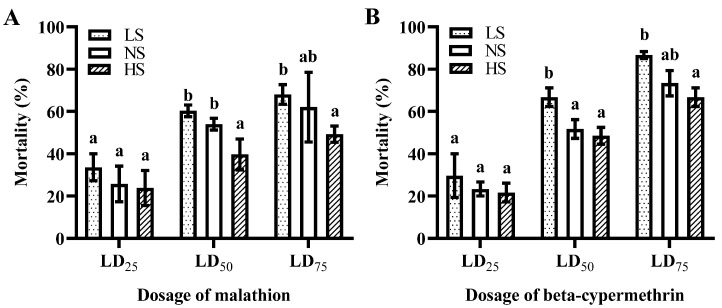
Mortality of *Bactrocera dorsalis*-fed dietary sucrose under different insecticide exposures. (**A**) Mortality of *B. dorsalis* under different concentrations of malathion; (**B**) Mortality of *B. dorsalis* under different concentrations of beta-cypermethrin. NS, normal sucrose; LS, low-sucrose; HS, high-sucrose. Bars with different letters differ significantly after analysis by one-way ANOVA (Tukey’s HSD tests, *p* < 0.05).

The mortality of various dosages of two insecticides against *B. dorsalis* adults was determined after their corresponding larvae were supplied with diets containing different concentrations of sucrose (Figure 2). The mortality of the HS group (*F* = 14.778; df = 2, 8; *p* = 0.005) was significantly lower than that in NS group under the treatment of malathion at LD_50_. However, there was no significant difference in the mortality under LD_25_ and LD_75_ between the LS or HS and NS groups (Figure 2A). On the contrary, the mortality of the LS group (*F* = 6.686; df = 2, 8; *p* = 0.034) was significantly higher than that of the NS group under the treatment of beta-cypermethrin at LD_50_. The same as for malathion, there were no significant differences in the mortality of LD_25_ and LD_75_ between each of the treatments and the NS group (Figure 2B).

### 3.4. Transcriptome Assembly and Differentially Expressed Genes (DEGs) Analysis

Comparing the clean reads to the genome sequences of *B. dorsalis*, the error rate of sequencing reads was between 0.0246 and 0.0254% for the tested samples (Table 2). In total, 71.48 GB of clean data was obtained, with an average Q20 of 98.09% and a Q30 of 94.15% (Table 2). In total, 15,212 genes were detected in the whole insects from 9 samples of *B. dorsalis* under different sucrose concentration exposures, and there were 13,843 (91.00%) genes that could be identified based on genome data. The remaining 1369 unidentified genes were viewed as novel genes.

**Figure 3:**
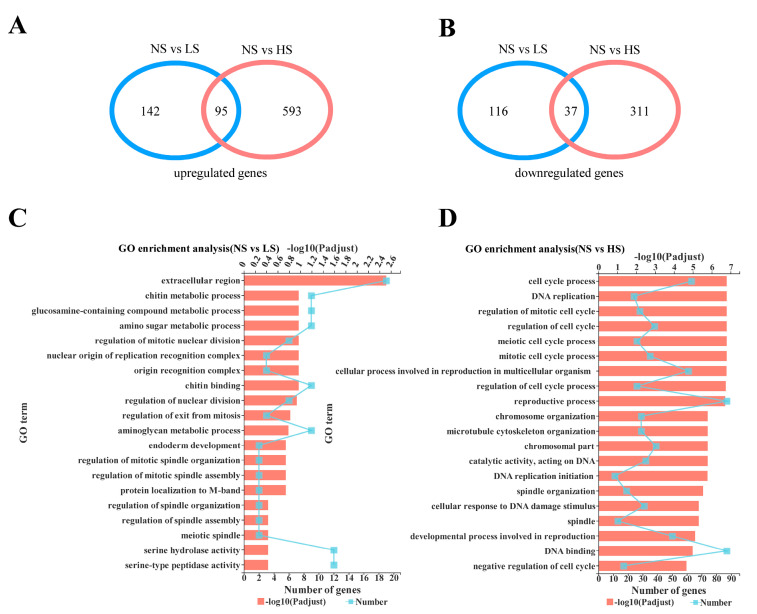
Differentially expressed genes (DEGs) analysis and gene ontology (GO) functional classification of the DEGs in *Bactrocera dorsalis*. (**A**) Venn diagram indicating the number of upregulated DEGs in the NS vs LS group and the NS vs HS group; (**B**) Venn diagram indicating the number of downregulated DEGs in the NS vs LS group and the NS vs HS group; (**C**) GO enrichment analysis of DEGs from NS vs LS group; (**D**) GO enrichment analysis of DEGs from NS vs HS group. NS, normal sucrose; LS, low-sucrose; HS, high-sucrose; vs, versus.

A total of 258 differentially expressed genes, comprising 116 down-regulated (44.96%) and 142 up-regulated (55.04%), were identified between the NS and LS groups (Figure 3A,B). In particular, the expression levels of *CYP313a4* (LOC105227996), *CYP304a1* (LOC105231048), *CYP6t1* (LOC105233159), and *CarE6* (LOC105229968) were down-regulated by 26.47-, 5.24-, 4.68-, and 3.99-fold, respectively (Figure S1). In the NS versus HS group, there were 904 DEGs, including 311 (34.40%) down-regulated and 593 (65.60%) up-regulated genes (Figure 3A,B). For example, in HS groups, the expressions levels of *CYP6a14* (LOC105228039), *CYP12b1* (LOC105226219), *CYP18a1* (LOC105224924), *CYP315a1* (LOC105232707), *GST1* (LOC105229686), and *CYP307a1* (LOC105225301) were significantly increased by 7.49-, 7.28-, 5.23-, 5.17-, 4.84-, and 3.11-fold when compared to the NS group, respectively (Figure S2). Additionally, 132 genes were both differentially expressed in the NS versus LS group and the NS versus HS group (Figure 3A,B).

### 3.5. Functional Annotation and Validation of DEGs

In the GO analysis, the DEGs were enriched into three ontologies, and the results are shown in Figure 3C,D. Interestingly, the “amino sugar metabolic process” (1.03%), “chitin binding” (0.99%), and “chitin metabolic process” (0.95%) terms were significantly enriched in the NS versus LS groups (Table S3). The expression level of *Cht10* (LOC105232357) in the LS group was down-regulated by 7.71-fold compared to the NS control group (Figure S1). We also found that the “reproductive process” (6.29%), “chromosome organization” (1.33%), and “spindle organization” (0.63%) terms were significantly enriched in the NS versus the HS group (Table S4). The expression of *Vm26Aa* (LOC105234031), *Vg1* (LOC105232170), and *Vg2* (LOC105222970) from the HS group were increased by 866.49-, 81.28-, and 5.85-fold compared to the NS control group, respectively (Figure S2).

In the KEGG analysis, the DEGs could be enriched into six categories, and the up-regulated pathways in NS versus LS group included “Toll and IMD signaling pathway” (1.23%), “Oocyte meiosis” (1.15%), “Folate biosynthesis” (0.59%), “Galactose metabolism” (0.52%), “Starch and sucrose metabolism” (0.49%), and “Thiamine metabolism” (0.30%) (Table S5). The up-regulated pathways between NS and HS groups also included “Oocyte meiosis” (1.15%), “Progesterone-mediated oocyte maturation” (0.83%), and “Fanconi anemia pathway” (0.40%). Importantly, the FoxO signaling pathway (0.97%) was also significantly enriched (Table S6). The down-regulated pathways in the NS versus LS group included “PI3K-Akt signaling pathway” (1.49%), “Protein digestion and absorption” (1.16%), “Insulin resistance” (0.82%), “Complement and coagulation cascades” (0.07%), and “Staphylococcus aureus infection” (0.03%) (Table S7). Meanwhile, the down-regulated pathways in the NS versus HS group consisted of “Lysosome” (1.72%), “Toll and IMD signaling pathway” (1.23%), and “Staphylococcus aureus infection” (0.03%), but were especially strong in “Glucagon signaling pathway” (0.95%) and “Growth hormone synthesis, secretion, and action” (0.92%) (Table S8).

**Figure 4:**
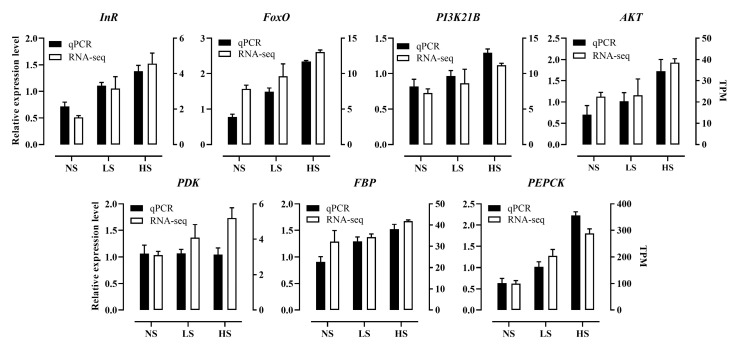
Relative expression levels of 7 selected genes in 5-d-old adults using qPCR. *x*-axis: 8.0% sucrose (low-sucrose, LS), 12.0% sucrose (normal sucrose, NS), and 16.0% sucrose (high-sucrose, HS). The left *y*-axis: the relative expression level. The right *y*-axis: the calculated TPM by RNA-seq (mean ± SE; *n* = 3).

Additionally, the qPCR data of 7 DEGs showed a similar expression pattern to the RNA-seq data (Figure 4), demonstrating the reliability of our data and analysis.

## 4. Discussion

For holometabolous insects, the larval stage is critical, and the nutrients obtained during this stage are directly related to subsequent individual growth and development [23]. In this study, we found that high sucrose concentrations in the larval diets resulted in the extension of the development duration of the immature stage (e.g., the larval and pupal periods) of *B. dorsalis*. Our results are somewhat consistent with previous research that has shown that diets with increased sugar content prolong the development period, and reduce the egg-to-adult survival rate and female fecundity of *Drosophila* species [3,24,25]. High sugar feeding induces systemic metabolic changes associated with carbohydrate metabolic imbalance, resulting in delayed development and reduced fresh weight in *Drosophila* [26,27]. On the contrary, some studies have shown that insect larvae will shorten their lifespan to avoid insufficient dietary sucrose conditions, and they usually emerge as smaller body size adults earlier [28]. The reason behind this phenomenon may be explained by the fact that low concentrations of sucrose cause abnormal glucose metabolism in insects, which shortens their developmental period [29]. Furthermore, the pre-oviposition period of female adults on the high-sucrose larval diet is obviously longer than that on the normal sucrose larval diet.

In many insects, vitellogenesis is a nutrient-dependent process that is promoted by the juvenile hormone (JH) [30,31]. We discovered that high sucrose intake during the larval stage has an effect on ovarian development during the adult stage, and that ovarian development is significantly delayed. However, lower sucrose intake in larvae does not affect adults’ reproductive organ development (Figure 1). Previous studies indicated that changes in dietary sucrose had an important influence on oxidative damage and oxidative stress in insects [7,32]. Under high-sucrose conditions, excessive sugar intake may result in insufficient glucose metabolism by *B. dorsalis*, which subsequently produces large amounts of reactive oxygen species (ROS). We speculated that the increase in sucrose metabolism in the early stage of *B. dorsalis* under high glucose stress restricted the development of the ovary by affecting the antioxidant system.

Interestingly, after higher sucrose consumption at the larval stage, the egg-laying capacity of *B. dorsalis* female adults was significantly higher than that in the control (NS) groups (Figure 1B). Therefore, the high sucrose intake in the larval stage enhanced the fecundity in the adult stage of *B. dorsalis*. We also found that the genes (*Vm26Aa*, *Vg1*, and *Vg2*) related to vitellogenesis and the genes (*CYP315a1*/*shadow* and *CYP307a1*/*spook*) associated with ecdysone synthesis were up-regulated in the adult stage in the transcriptomic data. Among these genes, *Vm26Aa*, *Vg1*, and *Vg2* were three key genes that played important roles in vitellogenesis and oocyte development [33,34]. Here, we inferred that high-sucrose diet intake in the larval stage may enhance reproductive capacity by increasing the expression of the above genes in their corresponding adult stages. Our results indicated that a good nutrition supply in the larval stage will play an important role in promoting the fecundity of the adult stage, but there is a balance between the ovarian development time and egg-laying amount.

In this study, relatively high- and low-sucrose larval diets altered the sensitivity of *B. dorsalis* adults to insecticides. Intriguingly, our results showed that high sucrose concentration consumption in larvae could significantly reduce the sensitivity of *B. dorsalis* to the LD_50_ of malathion (Figure 2A). However, low sucrose consumption in larvae can significantly increase the sensitivity of *B. dorsalis* adults to the LD_50_ of beta-cypermethrin (Figure 2B). Generally, bioassay data indicated that the adult flies formed by the larvae of the HS group have enhanced tolerance levels to insecticides when compared to the NS and LS groups (Figure 2). In *B. dorsalis*, resistance mechanisms may involve complex interactions of various genes, such as the detoxification enzymes, i.e., cytochrome P450s (P450s), glutathione S-transferases (GSTs), and carboxylesterases (CarEs) [35]. Based on transcriptomic data, we found some detoxification genes (e.g., *CYP6a14*, *CYP307a1*, and *GST1*) up-regulated in adult flies with their larvae fed a high-sucrose diet. The function of these above paralogous genes was confirmed to be associated with insecticide tolerance. For example, the knockdown of *CYP6A14-1*, *CYP307A1*, and *GST1-1-1* in a resistant aphid strain by RNAi resulted in increased susceptibility to insecticides [36]. Additionally, some more up-regulated detoxification genes, i.e., three P450 genes (*CYP313a4*, *CYP304a1*, *CYP6t1*) and one carboxylesterase gene (*CarE6*), may also contribute to the tolerance of insecticide in *B. dorsalis* adults after their larvae were exposed to high-sucrose diets. The expression level of *BdCYP437A3* was significantly up-regulated after 36 h of avermectin treatment [37]. Furthermore, CYP6 family genes are involved in the metabolism of highly effective beta cypermethrin [38]. Indeed, the genes *CYP304a1* and *CarE6* have been linked to the detoxification of multiple insecticides in insects [39,40,41].

In the NS vs. LS, DEGs were significantly enriched for “chitin metabolic process” and “chitin binding” terms, suggesting that *B. dorsalis* could adapt to the changes in sucrose content by regulating the expression of chitin metabolism-related and binding activity genes, which might be involved in the ability of *B. dorsalis* to cope with insecticide susceptibility. Indeed, cuticular alterations can also affect the intensity and spectrum of insecticide resistance or tolerance [42,43]. In this study, the chitin-related genes from the LS group were down-regulated, especially *Cht10*, and this result indicated that penetration resistance might be one of the important factors that resulted in the decreased insecticide tolerance of the LS group to exogenous harmful substances, i.e., beta-cypermethrin.

## 5. Conclusions

In summary, our work revealed many considerable differences in life history traits, fecundity, insecticide tolerance, and gene expression level associated with different sucrose concentrations of larval diets, which indicate that the phenotypic plasticity, reproduction-tolerance trade-offs, and gene expression of *B. dorsalis* are varied in response to the different dietary environments. These data also suggest that *B. dorsalis* can respond to food sucrose content by regulating developmental duration and fecundity, and inappropriate sucrose levels in the larval diet will also alter the tolerance of subsequent adult flies to insecticides. The findings of this study will contribute to a better understanding of the effect of sucrose on phenotypic adjustments, and the biological mechanism of rapid adaptation to new hosts in *B. dorsalis*.

## Figures and Tables

**Table 1:** The effects of different concentrations of sucrose in larval diets on the biological parameters of *Bactrocera dorsalis*.

Biological Parameters	Sucrose Concentration		
	8% (LS)	12% (NS)	16% (HS)
Larval Period (d)	10.62 ± 1.306 B	11.82 ± 1.705 A	12.24 ± 1.337 A
Pupation Rate (%)	63.33 ± 5.204 B	82.50 ± 9.014 A	80.83 ± 5.774 A
Pupal Period (d)	10.41 ± 1.501 A	11.07 ± 1.450 A	11.21 ± 1.325 A
Pupal Weight (mg)	17.10 ± 1.778 B	17.74 ± 1.684 A	17.04 ± 1.478 B
Eclosion Rate (%)	88.23 ± 5.344 A	91.82 ± 4.078 A	90.58 ± 3.289 A
Preoviposition Period (d)	9.47 ± 1.134 B	10.13 ± 1.381 B	11.02 ± 1.289 A

Note: data in the table are marked with the same uppercase letter, indicating that the difference is not significant after analysis by one-way ANOVA (Tukey’s Honestly Significant Difference test, *p* < 0.05). NS, normal sucrose; LS, low-sucrose; HS, high-sucrose; d, days.

**Table 2:** Sequencing summary of the transcriptome of *Bactrocera dorsalis* under different contents of sucrose mediated by larval diets.

Sample	Raw Reads	Clean Reads	Error Rate (%)	Q20 (%)	Q30 (%)
HS1	7.73 GB	7.56 GB	0.0249	98.08	94.17
HS2	8.44 GB	8.25 GB	0.0251	98.02	93.99
HS3	8.44 GB	8.25 GB	0.0250	98.09	94.14
LS1	8.09 GB	7.88 GB	0.0247	98.16	94.36
LS2	7.86 GB	7.69 GB	0.0254	97.90	93.72
LS3	8.67 GB	8.46 GB	0.0248	98.15	94.31
NS1	8.13 GB	7.95 GB	0.0252	97.99	93.93
NS2	8.00 GB	7.81 GB	0.0248	98.15	94.30
NS3	7.78 GB	7.63 GB	0.0246	98.24	94.47

Note: Q20: The proportion of reads with quality value ≥ 20; Q30: The proportion of reads with quality value ≥ 30.

## Data Availability

The data presented in this study are available in the Supplementary Materials.

## Supplementary Materials

The following supporting information can be downloaded at: https://www.mdpi.com/article/10.3390/insects14050407/s1: Figure S1. Expression levels of down-regulated genes between the NS and LS groups in *Bactrocera dorsalis.* Figure S2. Expression levels of up-regulated genes between NS and HS groups in *Bactrocera dorsalis*. Table S1. Composition of experimental diets for *Bactrocera dorsalis* larvae. Table S2. The information on qPCR primers for selected genes in *Bactrocera dorsalis*. Table S3. The gene ontology (GO) enrichment analysis between NS and LS groups in *Bactrocera dorsalis*. Table S4. The gene ontology (GO) enrichment analysis between NS and HS groups in *Bactrocera dorsalis*. Table S5. The up-regulated pathways of KEGG enrichment analysis between NS and LS groups in *Bactrocera dorsalis.* Table S6. The up-regulated pathways of KEGG enrichment analysis between NS and HS groups in *Bactrocera dorsalis.* Table S7. The down-regulated pathways of KEGG enrichment analysis between NS and LS groups in *Bactrocera dorsalis.* Table S8. The down-regulated pathways of KEGG enrichment analysis between NS and HS groups in *Bactrocera dorsalis.*
