# Supplementary material for: High-Sucrose Diet Exposure on Larvae Contributes to Adult Fecundity and Insecticide Tolerance in the Oriental Fruit Fly, Bactrocera dorsalis (Hendel)
Source: Insects. 2023 Apr 24;14(5):407. doi: 10.3390/insects14050407 (PMC10231089; doi:10.3390/insects14050407)
Supplement: Supplementary file 1 [file insects-14-00407-s001.zip › insects-2331593-supplementary figures.pdf]

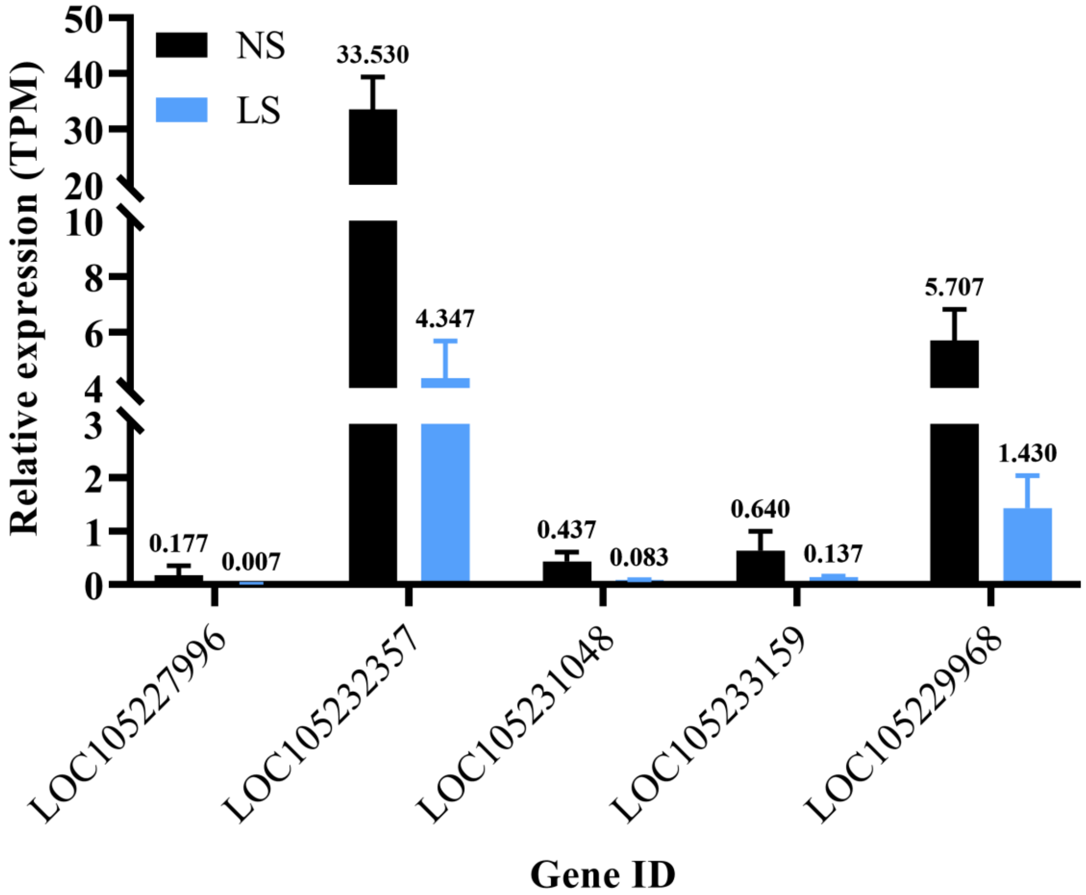


Figure S1. Expression levels of down-regulated genes between the NS and LS groups in *Bactrocera dorsalis*

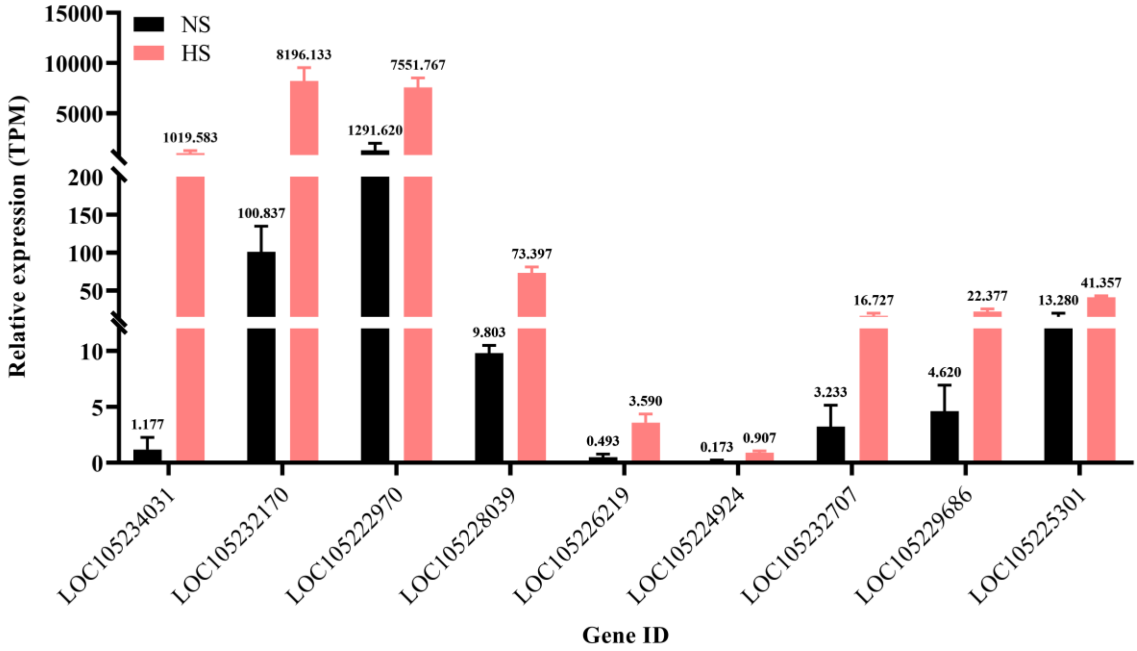


Figure S2. Expression levels of up-regulated genes between NS and HS groups in *Bactrocera dorsalis*
